# Associations of antibodies against citrullinated peptides with human leukocyte antigen-shared epitope and smoking prior to the development of rheumatoid arthritis

**DOI:** 10.1186/s13075-015-0638-x

**Published:** 2015-05-20

**Authors:** Heidi Kokkonen, Mikael Brink, Monika Hansson, Ewa Lassen, Linda Mathsson-Alm, Rikard Holmdahl, Johan Rönnelid, Lars Klareskog, Solbritt Rantapää-Dahlqvist

**Affiliations:** Institution of Public Health and Clinical Medicine/Rheumatolgy, Umeå University, SE-90185 Umeå, Sweden; Rheumatology Unit, Karolinska University Hospital, Stockholm, Sweden; Department of Immunology, Genetics and Pathology, Uppsala University, Uppsala, Sweden; Thermo Fisher Scientific, Uppsala, Sweden; Medical Inflammation Research, Department of Medical Biochemistry and Biophysics, Karolinska Institute, Stockholm, Sweden

## Abstract

**Introduction:**

It has previously been shown that an increased number of antibodies against citrullinated peptides/proteins (ACPA) predate the onset of rheumatoid arthritis (RA). Over time antibody positivity expands, involving more specific responses when approaching the onset of symptoms. We investigated the impact of human leukocyte antigen-shared epitope (HLA-SE) alleles and smoking on the development of ACPA, as well as in combination with ACPA during the state of quiescent autoimmunity (before the onset of symptoms), on the development of RA.

**Methods:**

Blood samples donated to the Medical Biobank of Northern Sweden from individuals prior to the onset of symptoms of RA (n = 370) and after onset (n = 203) and from population-based controls (n = 585) were used. Antibodies against 10 citrullinated peptides, fibrinogen (Fibα561-583, α580-600, ß62-81a, ß62-81b, ß36-52), vimentin (Vim2-17, 60-75), filaggrin (CCP-1/Fil307-324), α-enolase (CEP-1/Eno5-21), collagen type II (citC1359-369), and anti-cyclic citrullinated peptide (CCP)2 antibodies were analysed.

**Results:**

HLA-SE-positive individuals were more frequently positive for ACPA compared with HLA-SE-negative individuals prior to the onset of symptoms of RA, particularly for antibodies against CEP-1 and Fibß62-81a (72). Smoking was associated with antibodies against Vim2-17 and citC1359-369. HLA-SE and smoking showed increasing association to the presence of the antibodies closer to disease onset. The highest odds ratio (OR) for development of RA was for the combination of HLA-SE alleles and ACPA positivity, especially for antibodies against Fibß62-81b, CCP-1/Fil307-324, and Fibβ36-52. A gene-environment additive interaction between smoking and HLA-SE alleles for the risk of disease development was found, with the highest OR for individuals positive for antibodies against Fibβ36-52, CEP-1, and Fibα580-600.

**Conclusions:**

The relationships between antibodies against the different ACPA specificities, HLA-SE, and smoking showed a variable pattern in individuals prior to the onset of RA. The combination of smoking and HLA-SE alleles was significantly associated with the development of some of the antibody specificities closer to onset of symptoms, and these associations remained significant at diagnosis. An additive gene-environment interaction was found for several of the antibodies for the development of RA.

**Electronic supplementary material:**

The online version of this article (doi:10.1186/s13075-015-0638-x) contains supplementary material, which is available to authorized users.

## Introduction

Rheumatoid arthritis (RA) is a chronic autoimmune disease characterized by joint inflammation that eventually leads to the destruction of cartilage and bone. The aetiopathogenesis of the disease is not yet fully understood. It has previously been shown that the presence of antibodies against citrullinated proteins/peptides (ACPA), analysed as anti-cyclic citrullinated peptide (CCP) antibodies of immunoglobulin (Ig)G, IgA, and IgM isotypes, precedes the development of RA by several years [1-4]. More recently, we have also shown that an increased number of ACPA antibody specificities predate the onset of RA [5]. These ACPA antibody specificities were initially restricted and without any obvious epitope profile, but over time they expanded and involved more specific responses, especially antibodies against α-enolase (CEP-1/Eno5-21), fibrinogen (Fib)β36-52, and filaggrin (CCP-1/Fil307-324), when approaching the onset of symptoms [5].

The strongest genetic associations with RA known to date, HLA-shared epitope (SE) alleles and the protein tyrosine phosphatase non receptor type 22 (*PTPN22*) 1858C/T polymorphism, have been shown to be confined to ACPA-positive disease [6-8]. Recently, a study by Lundberg *et al*. showed that HLA-SE, *PTPN22* 1858T, and smoking were associated with specific ACPA reactivities, especially with antibodies against CEP-1 and citrullinated vimentin (anti-Vim 60-75) in patients with RA [9]. Additionally, in a study on Spanish RA patients, those with anti-CEP-1 antibodies showed an interaction with *PTPN22* 1858T and HLA-SE whilst having anti-cit-vimentin antibodies was associated with the presence of two HLA-SE alleles [10].

A recent study of genetic and environmental determinants for the development of ACPA in healthy twins as well as in twins with RA, demonstrated that smoking as well as HLA-SE alleles also influenced the development of ACPA in individuals without RA, although the impact of HLA-SE was less pronounced as risk factor for ACPA itself than for ACPA-positive RA [11]. The presence of reactivities against four different ACPA was associated with smoking and HLA-SE [11]. However, the role of the genetic factors, as well as of smoking, in the development of different ACPA specificities in individuals who have not yet developed RA, but will subsequently do so, have not yet been fully elucidated.

Interactions between a history of smoking and HLA-SE in the risk for seropositive RA has been demonstrated in several previous reports in early RA, the first one in 2006 [7], A modest additive interaction between a history of smoking and HLA-SE in the risk for development of seropositive RA was shown in analyses from the Nurses’ Health Study by Karlson *et al*. [12]. A strong additive interaction, as well as a significant multiplicative interaction, was found between the magnitude of past smoking and any HLA-SE alleles in the risk of seropositive RA [12]. In a continuation of that study, presence of multiple ACPA reactivities and of HLA-SE showed the highest risk for RA compared with ACPA-negative/HLA-SE-negative individuals as reference [13].

In the present study we aimed firstly to investigate the impact of HLA-SE alleles and smoking on the development of several ACPA specificities during the state of quiescent autoimmunity, that is, before the onset of symptoms, in individuals who all were known to later develop RA. Secondly we aimed to investigate the importance of these factors, independently and in combinations, in the development of RA. We have used blood samples donated to the Medical Biobank of Northern Sweden by these individuals prior to the onset of symptoms of RA and controls derived from the same population.

## Methods

A case-control study was conducted based on individuals included within the Medical Biobank of Northern Sweden and the Maternity cohort. The criteria for recruitment, collection, and storage of blood samples have been described previously [1].

The registers from the Medical Biobank and the Maternity cohort were co-analysed with the registers of patients with RA fulfilling the 1987 American Rheumatism Association classification criteria for RA [14], attending the Department of Rheumatology, University Hospital in Umeå, to identify individuals who had donated blood samples prior to onset of symptoms of RA. The number of individuals identified and the procedure for excluding any sample has previously been described in detail [5]. Originally, 386 individuals with a total of 717 plasma samples were identified as donors and were included in this study (71 men and 315 women, who were referred to as ‘pre-symptomatic individuals’). Of them, 370 individuals also had DNA available. In the present analyses, we used the sample from each individual that was the first that was positive for ACPA or anti-CCP2, respectively, if not presented differently. The median (IQR) time predating onset of symptoms was 4.6 (2.0 to 9.7) years. Controls (N = 1307) were randomly identified from the same cohorts within the register at the Medical Biobank and matched for age, sex, and date of sampling. Of these control subjects, 585 were randomly selected for genotyping and were included in this study. Of the pre-symptomatic individuals, 203 were also sampled when they were diagnosed with RA at the early arthritis clinic (median 7.2 months (IQR: 4.7 to 10.6 months) after symptom onset).

All individuals were classified either as a ‘non-smoker’ or an ‘ever-smoker’ (past or current). The Regional Ethics Committee at the University Hospital, Umeå, Sweden, approved this study, (Dnr 2011-168-31M) and all participants gave their written informed consent when donating blood samples. Demographic data for the pre-symptomatic individuals and controls are presented in Table 1.

**Table 1:** **Demographic data for individuals with samples at a median of 4.6 years (Q1 to Q3: 2.0 to 9.7) before the onset of symptoms of rheumatoid arthritis (RA) (pre-symptomatic individuals) at diagnosis and controls**

	**Pre-symptomatic individuals**	**RA**	**Controls** ^**a**^
	**(n = 370)**	**(n = 203)**	**(n = 585)**
Females (%)	81	77	71
Median age, years	49.9	56.5	50.3
(Q1-Q3) g	(30.6–58.8)	(48.7–63.7)	(40.2–60.1)
Ever-smoker (%)	230/348 (66.1)***	136/200 (68)***	254/552 (46.0)
HLA-DR SE^b^, n (%)	240/366 (65.6) ***	129/204 (63.2) ***	229/585 (39.1)
anti-CCP2 abs^c^, % (95% CI)	34.9 (30.2–39.9)***	74.5 (68.0–80.0)***^d^	2.4 (1.4–4.0)
anti-CEP-1/Eno5-21 abs, % (95% CI)	28.9 (24.5–33.7)***	67.3 (60.5–73.5)***^e^	6.1 (4.4–8.4)^f^
anti-citC1359-369 abs, % (95% CI)	13.1 (10–17.1)***	32.2 (26.1–39.0)***^e^	4.4 (3.0–6.4)^f^
anti-Fibα563-583 abs, % (95% CI)	10.8 (8–14.4)***	34.2 (27.9–41.0)***^e^	5.2 (3.6–7.4)^f^
anti-Fibα580-600 abs, % (95% CI)	8.7 (6.2–12.1)	14.1 (9.9–19.7)***^e^	6.1 (4.4–8.4)^f^
anti-Fibß36-52 abs, % (95% CI)	29.7 (25.3–34.6)***	64.8 (58.0–71.1)***^e^	6.6 (4.9–9.0)^f^
anti-Fibß62-81a (72) abs, % (95% CI)	11.6 (8.7–15.3)***	14.6 (10.3–20.2)***^e^	3.1 (2.0–5.0)^f^
anti-Fibß62-81b (74) abs, % (95% CI)	15.9 (12.5–20.1)***	34.2 (27.9–41.0)***^e^	1.7 (0.9–3.2)^f^
anti-CCP-1/Fil307-324 abs, % (95% CI)	26.2 (22–30.9)***	46.2 (39.4–53.2)***^e^	3.0 (1.8–4.7)^f^
anti-Vim60-75 abs, % (95% CI)	13.0 (9.9–16.8)***	29.1 (23.3–35.8)***^e^	5.4 (3.8–7.6)^f^
anti-Vim2-17 abs, % (95% CI)	7.0 (4.8–10.2)*	11.1 (7.4–16.3)***^e^	3.8 (2.5–5.8)^f^

*P <0.05, ***P <0.001 pre-symptomatic individuals or RA patients, respectively compared with controls.

^a^The sensitivity and specificity calculations are based on ROC curves performed originally on the RA cases (n = 200/199) and controls (n = 1,307) [5]. ^b^HLA-DR SE = shared epitope defined as 0101/0401/0404/0405/0408/. ^c^abs = antibodies. ^d^available number of samples analysed = 200. ^e^n = 199. ^f^n = 574. g (Q1-Q3)=first quartile to third quartile.

After the disease had developed and the individuals were diagnosed with RA, the frequency of antibodies against CCP2 was 75.2% using the anti-CCP2 test (see below).

### Multiplex assay

Samples were analysed for antibodies against the following 10 different citrullinated antigens: fibrinogen (Fib) α563-583, Fibα580-600, Fibβ62-81a (72), Fibβ62-81b (74), Fibβ36-52, α-Enolase (CEP-1/Eno5-21), triple helical collagen type II peptide containing the triple helical aa 359-369 with two citrulline residues (citC1359-369) [15], filaggrin (CCP-1/Fil307-324), vimentin (Vim) 2-17, and Vim60-75 using a custom-made microarray based on the ImmunoCAP ISAC® system (Phadia AB, Uppsala, Sweden), as previously described [5,16]. The difference in fluorescence intensity between the citrullinated peptide and the arginine-containing peptide was calculated and the resulting delta value was used. The exception was the citC1359-369 peptide, for which the uncorrected values were used [5,16]. The cutoff values for each antibody was set at the optimal level of sensitivity and specificity, calculated using receiver operator characteristic (ROC) curves as previously described [5]. A cutoff value for each of the antibodies was also tested at 97%, yielding similar frequencies of the antibodies as the ROC curves.

### Anti-CCP2 antibodies and HLA-SE alleles

Anti-CCP2 antibodies were detected using enzyme-linked immunosorbent assay (ELISA) according to the manufacturer’s instructions (Euro-Diagnostica AB, Malmö, Sweden). The cutoff level for positivity was set at 25 AU/mL. HLA-DRB1 0101/0401/0404/0405/0408 (SE alleles) were genotyped as described previously [17,18] (data presented in Table 1).

### Statistics

The chi-squared test, or Fisher’s exact test when appropriate, was used for testing categorical data (positive versus negative) between groups. Simple and multiple logistic regression analyses were performed to identify factors of importance for the development of antibodies against various citrullinated peptides and for the development of RA, and the risks are presented as odds ratios (OR) with 95% confidence intervals (CI). All *P* values are two-sided, and *P* values ≤0.05 are considered to be statistically significant. Statistical calculations were performed using SPSS for Windows version 20.0 (IBM SPSS, IBM Corp, NY, USA).

Analyses of interactions were tested for using the standard methods outlined in the article by Zou to calculate the attributable proportion (AP) due to interaction, the relative excess risk due to interaction (RERI), and the synergy index (SI) [19]. For models in which the variable HLA-SE was stratified into three categories (0, 1, or 2 HLA-SE alleles present), the additive interaction for each stratum was compared with a reference category of HLA-SE (no HLA-SE allele present). Multiplicative interaction was assessed by adding an interaction term (for example HLA-SE* smoking) to the regression models. A *P* value <0.05 was considered as evidence for departure from the multiplicative model of association. All the interactions were calculated using the statistical software R (R core team) [20].

## Results

### Impact of HLA-SE and smoking on the presence of different ACPA-fine specificities prior to the onset of symptoms and at diagnosis of rheumatoid arthritis

The presence of HLA-SE (defined as 0101/0401/0404/0405/0408) predicted positivity of antibodies against Fibß62-81a (72) (OR = 2.52, 95% CI: 1.13 to 5.61), and of CEP-1 (OR = 1.65, 95% CI: 1.003 to 2.73), including all pre-dating samples in the analyses (data not shown). Being homozygous for HLA-SE gave a higher OR for having antibodies against Fibß62-81b (74) (OR = 3.82, 95% CI: 1.57 to 9.32), Fibß62-81a (72) (OR = 3.24, 95% CI: 1.05 to 10.2), and CCP2 (OR = 2.41, 95% CI: 1.14 to 5.09) compared with being SE-allele-negative (data not shown). Individuals with HLA-SE had significantly more ACPA specificities, with a median number of antibodies of 1 (IQR: 3) compared with 0 (IQR: 1) for those who were HLA-SE negative, (*P* <0.01, data not shown). The conversion from being seronegative during the pre-symptomatic period and positive after disease onset was enhanced significantly by the presence of HLA-SE (OR = 3.3, 95% CI: 1.8 to 6.1). Being an ever-smoker among the pre-symptomatic individuals was associated with antibodies against Vim2-17 (OR = 4.05, 95% CI: 1.18 to 13.84), citC1359-369 (OR = 2.09, 95% CI: 1.00 to 4.39), and CCP2 (OR = 1.65, 95% CI: 1.02 to 2.66). However, smokers did not have a higher number of ACPA specificities compared with non-smokers (data not shown).

When all samples from the pre-symptomatic individuals were evaluated, the combination of HLA-SE alleles and smoking was significantly associated with antibodies against CCP2 and Fibβ62-81b, the reference being non-smoker without HLA-SE alleles (Table 2). When only including samples closer to disease onset in the analyses (when the frequencies of the different antibodies started to increase, ≤10.5 years prior to symptom onset of RA, n = 288 individuals), the presence of antibodies against CCP2, CEP-1/Eno5-21, citC1359-369, Fibβ62-81b, Fibβ36-52, and CCP-1/Fil307-324 were all significantly associated with the combination of HLA-SE and ever-smoking (Table 2). At the time of diagnosis, the combination of smoking and HLA-SE alleles had significant ORs for all of these antibodies, and additionally for Fibα561-583 and Vim60-75, with the exception of CCP-1/Fil307-324 that was non-significant after disease onset (Table 2).

**Table 2:** **Odds ratio for disease development of anti-CCP2 antibodies and different ACPA prior to symptoms of RA (pre-RA), ≤10.5 years prior to symptoms, and at diagnosis in combination with HLA-SE allele (none/any), smoking (never/ever)**

		**Pre-RA (ever-pos)**	**Pre-RA (≤10.5 years)**	**RA**
		**(n = 348)**	**(n = 277)**	**(n = 196)**
**HLA-SE**	**Smoking**	**OR (95% CI)**	**OR (95% CI)**	**OR (95% CI)**
**Anti-CCP2**				
None	Never	1.00 (Ref)	1.00 (Ref)	1.00 (Ref)
Any	Never	1.36 (0.57–3.22)	1.81 (0.81–4.00)	1.06 (0.36–3.14)
None	Ever	1.50 (0.64–3.53)	1.41 (0.62–3.22)	1.02 (0.36–2.88)
Any	Ever	**2.36 (1.08–5.17)**	**3.96 (1.87–8.39)**	**4.23 (1.42–12.59)**
**Anti-CEP-1/Eno5-21**				
None	Never	1.00 (Ref)	1.00 (Ref)	1.00 (Ref)
Any	Never	1.40 (0.56–3.55)	1.08 (0.46–2.55)	1.44 (0.51–4.03)
None	Ever	1.38 (0.55–3.48)	1.38 (0.59–3.27)	1.92 (0.70–5.26)
Any	Ever	2.25 (0.97–5.23)	**2.85 (1.30–6.24)**	**6.59 (2.42–17.99)**
**Anti-CitC1359-369**				
None	Never	1.00 (Ref)	1.00 (Ref)	1.00 (Ref)
Any	Never	1.20 (0.29–4.92)	1.74 (0.47–6.54)	3.88 (0.44–34.48)
None	Ever	2.00 (0.52–7.64)	2.28 (0.61–8.47)	**14.93 (1.85–120.33)**
Any	Ever	2.59 (0.74–9.07)	**4.05 (1.19–13.82)**	**14.67 (1.89–113.98)**
**Anti-Fibα580-600 (591)**				
None	Never	1.00 (Ref)	1.00 (Ref)	1.00 (Ref)
Any	Never	1.31 (0.38–4.47)	1.57 (0.41–5.96)	0.17 (0.02–1.75)
None	Ever	0.61 (0.15–2.40)	0.88 (0.20–3.83)	1.37 (0.33–5.73)
Any	Ever	0.69 (0.20–2.26)	1.39 (0.38–5.07)	1.32 (0.34–5.03)
**Anti-Fibα563-583 (573)**				
None	Never	1.00 (Ref)	1.00 (Ref)	1.00 (Ref)
Any	Never	1.61 (0.31–8.34)	1.89 (0.39–9.21)	2.22 (0.54–9.09)
None	Ever	2.79 (0.58–13.42)	3.16 (0.67–14.84)	3.13 (0.80–12.17)
Any	Ever	3.09 (0.69–13.79)	3.42 (0.77–15.17)	**5.14 (1.42–18.61)**
**Anti-Fibβ62-81a (72)**				
None	Never	1.00 (Ref)	1.00 (Ref)	1.00 (Ref)
Any	Never	**4.98 (1.09–22.90)**	4.90 (0.61–39.36)	2.44 (0.26–23.30)
None	Ever	1.58 (0.31–8.23)	3.33 (0.39–28.40)	1.50 (0.15–15.27)
Any	Ever	2.76 (0.61–12.36)	6.99 (0.91–53.38)	5.91 (0.75–46.86)
**Anti-Fibβ62-81b (74)**				
None	Never	1.00 (Ref)	1.00 (Ref)	1.00 (Ref)
Any	Never	2.00 (0.52–7.64)	7.14 (0.91–55.83)	2.53 (0.63–10.23)
None	Ever	2.00 (0.52–7.64)	6.45 (0.81–51.41)	2.55 (0.65–10.05)
Any	Ever	**3.52 (1.02–12.20)**	**16.73 (2.24–124.88)**	**5.93 (1.64–21.44)**
**Anti-Fibβ36-52**				
None	Never	1.00 (Ref)	1.00 (Ref)	1.00 (Ref)
Any	Never	1.20 (0.50–2.86)	1.45 (0.58–3.67)	0.85 (0.30–2.39)
None	Ever	1.18 (0.49–2.81)	1.65 (0.65–4.20)	0.80 (0.29–2.19)
Any	Ever	1.64 (0.74–3.61)	**3.64 (1.54–8.57)**	**3.08 (1.15–8.21)**
**Anti-CCP-1/Fil307-324**				
None	Never	1.00 (Ref)	1.00 (Ref)	1.00 (Ref)
Any	Never	1.81 (0.73–4.51)	**3.67 (1.34–10.06)**	0.66 (0.23–1.85)
None	Ever	1.20 (0.47–3.06)	1.97 (0.68–5.69)	0.51 (0.18–1.42)
Any	Ever	1.77 (0.76–4–14)	**4.05 (1.52–10.79)**	1.42 (0.56–3.56)
**Anti-Vim60−75**				
None	Never	1.00 (Ref)	1.00 (Ref)	1.00 (Ref)
Any	Never	1.42 (0.47–4.32)	6.56 (0.84–51.59)	2.53 (0.63–10.23)
None	Ever	0.58 (0.17–2.04)	2.74 (0.31–24.11)	1.17 (0.27–5.00)
Any	Ever	1.02 (0.35–2.91)	6.20 (0.81–47.64)	**4.67 (1.29**–**16.92)**
**Anti-Vim2-17**				
None	Never	1.00 (Ref)	1.00 (Ref)	1.00 (Ref)
Any	Never	1.04 (0.09–11.83)	2.34 (0.27–20.54)	1.41 (0.33–6.10)
None	Ever	2.67 (0.30–23.68)	2.17 (0.24–19.94)	0.62 (0.13–3.04)
Any	Ever	4.99 (0.64–38.65)	6.20 (0.81–47.64)	0.69 (0.17–2.85)

Significant associations presented in bold. HLA-SE= HLA shared epitope, OR= odds ratio, CI= confidence intervals, anti-CCP2=anti-cyclic citrullinated peptide antibodies, anti CEP-1/Eno5-21= antibodies against α-Enolase, anti-citC1359-369= antibodies against collagen type II aa 359-369 with two citrulline residues, anti- Fibα580-600=antibodies against fibrinogen (Fib) Fibα580-600, anti-Fibα561-583= antibodies against Fibα561-583, anti- Fibβ62-81a(72)=antibodies against Fibβ62-81a(72), anti-Fibβ62-81b(74)= antibodies against Fibβ62-81b(74), anti-Fibβ36-52= antibodies against Fibβ36-52, anti- CCP-1/Fil307-324= antibodies against filaggrin, anti- Vim60-75= antibodies against vimentin (Vim)60-75, anti-Vim2-17= antibodies against Vim 2-17.

Further analyses of the association of HLA-SE alleles with the levels of the different antibodies showed that higher levels of antibodies (three times above the cutoff level for each antibody) against CCP2, CEP-1/Eno5-21, and Fibß62-81b was associated with having HLA-SE alleles (*P* <0.05 for all, data not shown).

### Impact of HLA-SE, smoking, and individual ACPA prior to onset of symptoms on the risk of development of rheumatoid arthritis

All ACPA analysed, except anti-Fibα580-600, predicted the development of RA (Table 3). Adjustment of the data for smoking and HLA-SE did not affect the calculated risk of the antibodies, except that anti-Vim2-17 antibodies became non-significant when adjusted for smoking (Table 3).

**Table 3:** **Risk for development of RA in individuals before the onset of any symptoms of disease (n = 370)**

**Antibodies**	**Frequency**	**OR (95% CI)**	**OR (95% CI)**	**OR (95% CI)**
	**N (%)**		**Adjusted for smoking**	**Adjusted for HLA-SE**
			**(n = 348)**	**(n = 366)**
Anti-CCP2	129 (34.9)	21.83 (12.33–38.67)	21.69 (11.96–39.33)	19.52 (10.96–34.79)
Anti-CEP-1/Eno5-21	107 (28.9)	6.27 (4.16–9.43)	5.69 (3.74–8.66)	5.57 (3.67–8.46)
Anti-CitC1359-369	47 (13.1)	3.32 (2.00–5.50)	3.04 (1.82–5.10)	3.27 (1.94–5.50)
Anti-Fibα580-600 (591)	31 (8.7)	1.46 (0.88–2.41)	1.39 (0.82–2.36)	1.54 (0.91–2.61)
Anti-Fibα563-583 (573)	40 (10.8)	2.20 (1.34–3.60)	2.10 (1.25–3.53)	2.15 (1.29–3.57)
Anti-Fibβ62-81a (72)	43 (11.6)	4.06 (2.30–7.16)	4.42 (2.47–7.91)	3.44 (1.92–6.17)
Anti-Fibβ62-81b (74)	57 (15.9)	10.68 (5.38–21.22)	10.05 (5.02–20.10)	9.48 (4.72–19.05)
Anti-Fibβ36-52	110 (29.7)	5.97 (4.01–8.88)	6.07 (4.00–9.22)	5.66 (3.76–8.51)
Anti-CCP-1/Fil307-324	97 (26.2)	11.64 (6.82–19.88)	12.10 (6.94–21.10)	10.39 (6.03–17.90)
Anti-Vim60-75	48 (13.0)	2.61 (1.63–4.19)	2.37 (1.45–3.88)	2.50 (1.54–4.08)
Anti-Vim2-17	29 (7.0)	1.90 (1.06–3.40)	1.55 (0.85–2.85)	1.85 (1.01–.3.39)

OR= odds ratio, CI= confidence intervals, anti-CCP2=anti-cyclic citrullinated peptide antibodies, anti CEP-1/Eno5-21= antibodies against α-Enolase, anti-citC1359-369= antibodies against collagen type II aa 359-369 with two citrulline residues, anti- Fibα580-600=antibodies against fibrinogen (Fib) Fibα580-600, anti-Fibα561-583= antibodies against Fibα561-583, anti- Fibβ62-81a(72)=antibodies against Fibβ62-81a(72), anti-Fibβ62-81b(74)= antibodies against Fibβ62-81b(74), anti-Fibβ36-52= antibodies against Fibβ36-52, anti- CCP-1/Fil307-324= antibodies against filaggrin, anti- Vim60-75= antibodies against vimentin (Vim)60-75, anti-Vim2-17= antibodies against Vim 2-17.

In the pre-symptomatic individuals HLA-SE, and particularly HLA-SE homozygosity was associated with subsequent development of RA (OR = 2.96, 95% CI: 2.26 to 3.89 and OR = 5.47, 95% CI: 3.05 to 9.83, respectively). Ever smoking was similarly associated with disease development (OR = 2.29, 95% CI: 1.73 to 3.02; data not shown).

### Relationship of HLA-SE alleles and ACPA reactivities in development of rheumatoid arthritis

**Figure 1:**
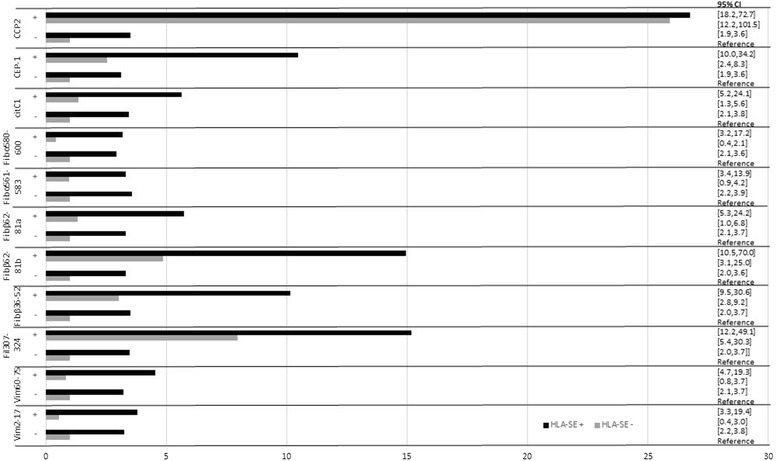
Odds ratio (OR) for development of RA in individuals prior to the onset of RA stratified for ACPA reactivity and the presence of HLA-SE alleles. Individuals without HLA-SE and ACPA were used as reference. HLA-SE+= HLA shared epitope present, HLA-SE-=HLA shared epitope absent, OR= odds ratio, CI= confidence intervals, anti-CCP2=anti-cyclic citrullinated peptide antibodies, anti CEP-1/Eno5-21= antibodies against α- Enolase, anti-citC1359-369= antibodies against collagen type II aa 359-369 with two citrulline residues, anti- Fibα580-600=antibodies against fibrinogen (Fib) Fibα580-600, anti-Fibα561-583= antibodies against Fibα561-583, anti- Fibβ62-81a(72)=antibodies against Fibβ62-81a(72), anti-Fibβ62-81b(74)= antibodies against Fibβ62-81b(74), anti-Fibβ36-52= antibodies against Fibβ36-52, anti- CCP-1/Fil307-324= antibodies against filaggrin, anti- Vim60-75= antibodies against vimentin (Vim)60-75, anti-Vim2-17= antibodies against Vim 2-17.

The highest risk for development of RA was to be positive for the combination of HLA-SE and ACPA, using individuals lacking both HLA-SE and ACPA as reference. The highest OR was found for the combinations of HLA-SE alleles and antibodies against CCP2, Fibβ62-81b, CCP-1/Fil307-324, CEP-1/Eno5-21, and Fibß36-52 (Figure 1). It is also evident that the impact of the presence of HLA-SE varies strongly between the different antibodies. This analysis was also performed in the ACPA-positive subgroup (including cases and controls positive for any ACPA reactivity), to exclude the possibility that the interactions are explained simply by ACPA status, and yielded similar results.

### Combinations of separate or several ACPA with smoking and HLA-SE alleles on the risk of development of rheumatoid arthritis

Individuals were distributed into subgroups based on the presence or absence of ever smoking, and carriage of HLA-SE alleles and of individual ACPA or ACPA combinations, with the reference groups comprising of ACPA-negative non-smokers without HLA-SE. The combinations of being a smoker and having HLA-SE together with any of the four most frequently occurring antibodies, (CEP-1/Eno5-21, Fibß36-52, CCP-1/Fil307-324, and CCP2) yielded very high ORs in relation to HLA-SE- and ACPA-negative non-smokers, although these combinations of triplets comprised relatively low numbers of individuals (presented in Table 4). The combination of three (CEP-1/Eno5-21, Fibß36-52, and CCP-1/Fil307-324) or four (CCP2, CEP-1/Eno5-21, Fibß36-52, and CCP-1/Fil307-324) of the most frequently occurring antibodies being positive together with smoking and HLA-SE resulted in even higher ORs for development of RA among the pre-symptomatic individuals (OR = 61.03, 95% CI: 13.95 to 267.05), compared with being negative for any of the antibodies (Table 4). Analysing for all four antibodies (CCP2, CEP-1/Eno5-21, Fibß36-52, and CCP-1/Fil307-324) being positive versus all four being negative yielded an even higher OR (Table 4). Multiple logistic regression analyses including each separate ACPA and anti-CCP2 antibodies, HLA-SE, and smoking showed that all three remained significant as independent predictors for the development of RA in all analyses (Additional file 1: Table S1).

**Table 4:** **Odds ratios (ORs) for disease development in pre-symptomatic individuals for different ACPA and anti-CCP2 in combination with smoking and HLA-SE compared with individuals being ACPA/anti-CCP2 negative, HLA-SE negative, and non-smokers as reference**

**Anti-CCP2/ACPA**	**Smoking**	**HLA-SE**	**Cases/controls**	**OR (95% CI)**
	**(n = 348)**	**(n = 366)**	**n/n**	
**Anti-CCP2**			345/552^a^	
−	−	−	31/172	Ref
+	−	−	10/1	55.48 (6.86–448.99)
−	+	−	52/160	1.80 (1.10–2.96)
–	–	+	52/121	2.38 (1.44–3.94)
+	+	–	26/3	48.09 (13.71–168.64)
−	+	+	84/86	5.42 (3.33–8.82)
+	−	+	24/4	33.29 (10.80–102.59)
+	+	+	66/5	73.24 (27.32–196.37)
**Anti-CEP-1/Eno5-21**			345/541^a^	
−	−	−	33/163	Ref
+	−	−	8/7	5.65 (1.92–16.64)
−	+	−	58/147	1.95 (1.20–3.16)
−	−	+	56/117	2.36 (1.45–3.86)
+	+	−	20/14	7.05 (3.24–15.37)
−	+	+	96/79	6.00 (3.72–9.68)
+	−	+	20/7	14.11 (5.52–36.08)
+	+	+	54/7	38.10 (15.94–91.11)
**Anti-Fibβ36-52**			345/541^a^	
**−**	−	−	31/161	Ref
+	−	−	10/9	5.77 (2.17–15.36)
−	+	−	56/149	1.95 (1.19–3.19)
−	−	+	54/117	2.40 (1.45–3.96)
+	+	−	22/12	9.52 (4.27–21.22)
−	+	+	97/79	6.38 (3.92–10.37)
+	−	+	22/7	16.32 (6.42–41.51)
**+**	+	+	53/7	39.32 (16.36–94.51)
**Anti-CCP-1/Fil307-324**			345/541^a^	
**−**	−	−	33/168	Ref
+	−	−	8/2	20.36 (4.14–100.24)
−	+	−	60/157	1.95 (1.21–3.14)
−	−	+	52/120	2.21 (1.35–3.62)
+	+	−	18/4	22.91 (7.28–72.05)
−	+	+	104/80	6.62 (4.12–10.63)
+	−	+	24/4	30.55 (9.94–93.84)
**+**	+	+	46/6	39.03 (15.42–98.82)
**Anti-CEP-1/Eno5-21 + anti-Fibβ36-52 + anti-CCP-1/Fil307-324** ^**b**^			345/541^a^	
−	−	−	39/170	Ref
+	−	−	1/0	−
−	+	−	72/159	1.97 (1.26–3.08)
−	−	+	66/123	2.33 (1.48–3.70)
+	+	−	6/1	26.15 (3.06–223.50)
−	+	+	122/85	6.26 (4.01)
+	−	+	11/1	47.95 (6.01–382.46)
+	+	+	28/2	61.03 (13.95–267.05)
**Anti-CEP-1/Eno5-21 + anti-Fibβ36-52 + anti-CCP-1/Fil307-324 + anti-CCP2** ^**c**^			204/464^a^	
−	−	−	20/151	Ref
+	−	−	1/0	−
−	+	−	37/133	2.10 (1.16–3.80)
−	−	+	37/105	2.66 (1.46–4.84)
+	+	−	5/0	−
−	+	+	65/72	6.82 (3.84–12.11)
+	−	+	11/1	83.05 (10.17–677.91)
+	+	+	28/2	105.70 (23.39–477.74)

^a^Total number included in analysis. ^b^Positive = positive for all of the three antibodies and negative = any of the antibodies negative. ^c^Positive = all four antibodies positive and negative = all four antibodies negative. anti-CCP2=anti-cyclic citrullinated peptide antibodies, ACPA= anti citrullinated peptide/protein antibodies, HLA-SE= HLA shared epitope, OR= odds ratio, CI= confidence intervals, anti CEP-1/Eno5-21= antibodies against alpha-Enolase, anti-citC1359-369= antibodies against collagen type II aa 359-369 with two citrulline residues, anti- Fibα580-600=antibodies against fibrinogen (Fib) Fibα580-600, anti-Fibα561-583= antibodies against Fibα561-583, anti- Fibβ62-81a(72)=antibodies against Fibβ62-81a(72), anti-Fibβ62-81b(74)= antibodies against Fibβ62-81b(74), anti-Fibβ36-52= antibodies against Fibβ36-52, anti- CCP-1/Fil307-324= antibodies against filaggrin, anti- Vim60-75= antibodies against vimentin (Vim)60-75, anti-Vim2-17= antibodies against Vim 2-17.

### Gene-environment interactions in individuals, prior to diagnosis, for risk of developing rheumatoid arthritis

An additive interaction was evident between smoking and HLA-SE alleles regardless of ACPA status in individuals prior to the diagnosis of RA, with an AP of 0.48 (95% CI: 0.43 to 0.55) (Table 5). When stratifying the data from individuals prior to the onset of symptoms of RA according to having the various ACPA specificities, a gene-environment additive interaction for development of RA was found for seropositivity as well as seronegativity; however, there was at least a doubled OR for ACPA-fine specificity-positive individuals, with the exception of anti-Fibα580-600 and anti-Vim60-75 antibodies (Table 5). There were no significant multiplicative gene-environment interactions of HLA-SE alleles and smoking in any of the groups for the development of RA among individuals prior to diagnosis. Also, when analysing the relationship of HLA-SE and the most frequent ACPA reactivities, an additive interaction for development of RA between HLA-SE and some of the ACPA was significant, namely for CEP-1/Eno5-21 (AP = 0.67, 95% CI: 0.57 to 0.73) and Fibß36-52 (AP = 0.60, 95% CI: 0.49 to 0.67), as was being positive for any of the ACPA (AP = 0.61, 95% CI: 0.58 to 0.65). No significant interactions were shown for smoking together with the ACPA reactivities or anti-CCP2 antibodies (data not shown). There were no significant interactions for the triple combination of presence of HLA-SE, smoking, and anti-CEP-1/Eno5-21, anti-CCP-1/Fil307-324, or anti-Fibß36-52 antibodies, or any ACPA separately analysed.

**Table 5:** **Gene-environment interaction of HLA-SE alleles and smoking in individuals prior to diagnosis of RA stratified for the presence of autoantibodies against various citrullinated peptides compared with controls**

		**All**			
**HLA-SE**	**Smoking**	**Cases/controls**	**OR (95% CI)**		
None	Never	41/173	1.00 (Ref)		
None	Ever	78/162	2.03 (1.32–3.14)		
Any	Never	76/125	2.57 (1.65–4.00)		
Any	Ever	150/92	6.88 (4.48–10.56)		
**Interaction**					
AP			0.48 (0.43–0.55)		
RERI			3.28 (1.43–6.16)		
SI			2.26 (1.36–3.76)		
MI			*P* = 0.35		
		**Anti-CCP2 positive**		**Anti-CCP2 negative**	
**HLA-SE**	**Smoking**	**Cases/controls**	**OR (95% CI)**	**Cases/controls**	**OR (95% CI)**
None	Never	10/173	1.00 (Ref)	31/173	1.00 (Ref)
None	Ever	26/162	2.77 (1.30–5.94)	52/162	1.79 (1.09–2.94)
Any	Never	24/125	3.32 (1.53–7.19)	52/125	2.32 (1.41–3.83)
Any	Ever	66/92	12.41 (6.09–25.28)	84/92	5.10 (3.14–8.26)
**Interaction**					
AP			0.59 (0.53–0.67)		0.39 (0.32–0.50)
RERI			7.31 (3.35–17.90)		1.98 (0.21–4.41)
SI			2.78 (1.49–5.19)		1.94 (1.03–3.66)
MI			*P* = 0.53		*P* = 0.55
		**Anti-CCP-1/Fil307-324 positive**		**Anti-CCP-1/Fil307-324 negative**	
**HLA-SE**	**Smoking**	**Cases/controls**	**OR (95% CI)**	**Cases/controls**	**OR (95% CI)**
None	Never	8/173	1.00 (Ref)	33/173	1.00 (Ref)
None	Ever	18/162	2.40 (1.02–5.68)	60/162	1.94 (1.21–3.13)
Any	Never	24/125	4.15 (1.81–9.55)	52/125	2.18 (1.33–3.57)
Any	Ever	46/92	10.81 (4.90–23.88)	104/92	5.93 (3.72–9.45)
**Interaction**					
AP			0.49 (0.40–0.60)		0.47 (0.41–0.56)
RERI			5.26 (0.52–15.71)		2.80 (1.02–5.55)
SI			2.15 (1.11–4.16)		2.32 (1.26–4.28)
MI			*P* = 0.88		*P* = 0.30
		**Anti-Fibβ36-52 positive**		**Anti-Fibβ36-52 negative**	
**HLA-SE**	**Smoking**	**Cases/controls**	**OR (95% CI)**	**Cases/controls**	**OR (95% CI)**
None	Never	10/173	1.00 (Ref)	31/173	1.00 (Ref)
None	Ever	22/162	2.35 (1.08–5.11)	56/162	1.93 (1.18–3.14)
Any	Never	22/125	3.04 (1.39–6.66)	54/125	2.41 (1.47–3.97)
Any	Ever	53/92	9.97 (4.84–20.51)	97/92	5.88 (3.65–9.48)
**Interaction**					
AP			0.56 (0.48–0.66)		0.43 (0.37–0.52)
RERI			5.57 (1.95–14.16)		2.54 (0.68–5.31)
SI			2.64 (1.31–5.34)		2.09 (1.15–3.77)
MI			*P* = 0.50		*P* = 0.48
		**Anti-CEP-1/Eno5-21 positive**		**Anti-CEP-1/Eno5-21 negative**	
**HLA-SE**	**Smoking**	**Cases/controls**	**OR (95% CI)**	**Cases/controls**	**OR (95% CI)**
None	Never	8/173	1.00 (Ref)	33/173	1.00 (Ref)
None	Ever	20/162	2.67 (1.14–6.23)	58/162	1.88 (1.16–3.03)
Any	Never	20/125	3.46 (1.47–8.11)	56/125	2.35 (1.44–3.83)
Any	Ever	54/92	12.69 (5.79–27.81)	96/92	5.47 (3.42–8.75)
**Interaction**					
AP			0.60 (0.52–0.69)		0.41 (0.34–0.51)
RERI			7.56 (3.14–20.18)		2.24 (0.47–4.77)
SI			2.83 (1.44–5.58)		2.00 (1.10–3.66)
MI			*P* = 0.54		*P* = 0.51
		**Anti-Fibβ62-81a (72) positive**		**Anti-Fibβ62-81a ( 72) negative**	
**HLA-SE**	**Smoking**	**Cases/controls**	**OR (95% CI)**	**Cases/controls**	**OR (95% CI)**
None	Never	2/173	1.00 (Ref)	39/173	1.00 (Ref)
None	Ever	6/162	3.20 (0.64–16.10)	72/162	1.97 (1.26–3.08)
Any	Never	16/125	11.07 (2.50–49.02)	60/125	2.13 (1.34–3.39)
Any	Ever	19/92	17.86 (4.07–78.38)	131/92	6.32 (4.08–9.79)
**Interaction**					
AP			0.26 (0.02–0.48)		0.51 (0.46–0.59)
RERI			4.59 (–32.49−55.67)		3.21 (1.47–5.96)
SI			1.37 (0.65–2.90)		2.53 (1.42–4.52)
MI			*P* = 0.45		*P* = 0.18
		**Anti-Fibβ62-81b (74) positive**		**Anti-Fibβ62-81b (74) negative**	
**HLA-SE**	**Smoking**	**Cases/controls**	**OR (95% CI)**	**Cases/controls**	**OR (95% CI)**
None	Never	3/173	1.00 (Ref)	37/173	1.00 (Ref)
None	Ever	11/162	3.92 (1.07–14.29)	66/162	1.90 (1.21–3.00)
Any	Never	11/125	5.07 (1.39–18.57)	65/125	2.43 (1.53–3.87)
Any	Ever	32/92	20.06 (5.98–67.28)	109/92	5.54 (3.53–8.69)
**Interaction**					
AP			0.60 (0.49–0.73)		0.40 (0.33–0.49)
RERI			12.07 (−0.05–53.96)		2.20 (0.47–4.63)
SI			2.72 (1.28–5.79)		1.94 (1.11–3.41)
MI			*P* = 0.99		*P* = 0.57
		**Anti-Fibα580-600 positive**		**Anti-Fibα580-600 negative**	
**HLA-SE**	**Smoking**	**Cases/controls**	**OR (95% CI)**	**Cases/controls**	**OR (95% CI)**
None	Never	4/173	1.00 (Ref)	36/173	1.00 (Ref)
None	Ever	5/162	1.34 (0.35–5.06)	72/162	2.14 (1.36–3.36)
Any	Never	10/125	3.46 (1.06–11.28)	66/125	2.54 (1.59–4.05)
Any	Ever	29/92	4.70 (1.44–15.40)	131/92	6.84 (4.38–10.70)
**Interaction**					
AP			0.72 (0.60–0.87)		0.46 (0.41–0.54)
RERI			9.84 (3.70–33.71)		3.17 (1.25–6.16)
SI			4.52 (1.37–14.87)		2.19 (1.30–3.67)
MI			*P* = 0.17		*P* = 0.45
		**Anti-citC1359-369 positive**		**Anti-citC1359-369 negative**	
**HLA-SE**	**Smoking**	**Cases/controls**	**OR (95% CI)**	**Cases/controls**	**OR (95% CI)**
None	Never	3/173	1.00 (Ref)	37/173	1.00 (Ref)
None	Ever	11/162	3.92 (1.07–14.29)	66/162	1.90 (1.21–3.00)
Any	Never	7/125	3.23 (0.82–12.73)	69/125	2.58 (1.63–4.09)
Any	Ever	25/92	15.67 (4.61–53.29)	116/92	5.89 (3.77–9.23)
**Interaction**					
AP			0.61 (0.46–0.77)		0.41 (0.35–0.49)
RERI			9.52 (−1.11–43.03)		2.41 (0.62–4.97)
SI			2.85 (1.16–6.98)		1.97 (1.14–3.39)
MI			*P* = 0.79		*P* = 0.56
		**Anti-Vim60-75 positive**		**Anti-Vim60-75 negative**	
**HLA-SE**	**Smoking**	**Cases/controls**	**OR (95% CI)**	**Cases/controls**	**OR (95% CI)**
None	Never	5/173	1.00 (Ref)	36/173	1.00 (Ref)
None	Ever	6/162	1.28 (0.38–4.28)	72/162	2.14 (1.36–3.36)
Any	Never	13/125	3.60 (1.25–10.35)	63/125	2.42 (1.51–3.87)
Any	Ever	19/92	7.14 (2.58–19.76)	131/92	6.84 (4.38–10.70)
**Interaction**					
AP			0.46 (0.24–0.70)		0.48 (0.42–0.55)
RERI			3.27 (−2.87–13.75)		3.28 (1.38–6.29)
SI			2.13 (0.72–6.36)		2.28 (1.34–3.88)
MI			*P* = 0.55		*P* = 0.37
		**Anti-Fibα563-583 positive**		**Anti-Fibα561-583 negative**	
**HLA-SE**	**Smoking**	**Cases/controls**	**OR (95% CI)**	**Cases/controls**	**OR (95% CI)**
None	Never	2/173	1.00 (Ref)	38/173	1.00 (Ref)
None	Ever	10/162	5.34 (1.15–24.74)	68/162	1.86 (1.19-2-92)
Any	Never	6/125	4.15 (0.82–20.91)	71/125	2.48 (1.58–3.91)
Any	Ever	21/92	19.74 (4.53–86.07)	129/92	6.22 (4.01–9.64)
**Interaction**					
AP			0.57 (0.39–0.76)		0.46 (0.41–0.54)
RERI			11.25 (−10.52–71.35)		2.87 (1.10–5.54)
SI			2.50 (1.05–5.95)		2.22 (1.29–3.85)
MI			*P* = 0.90		*P* = 0.33
		**Anti-Vim2-17 positive**		**Anti-Vim2-17 negative**	
**HLA-SE**	**Smoking**	**Cases/controls**	**OR (95% CI)**	**Cases/controls**	**OR (95% CI)**
None	Never	1/173	1.00 (Ref)	40/173	1.00 (Ref)
None	Ever	5/162	5.34 (0.62–46.20)	73/1162	1.95 (1.25–3.03)
Any	Never	2/125	2.77 (0.25–30.87)	74/125	2.56 (1.64–4.01)
Any	Ever	17/92	31.97 (4.19–244.04)	133/92	6.25 (4.05–9.66)
**Interaction**					
AP			0.77 (0.54–0.99)		0.47 (0.42–0.54)
RERI			24.86 (−16.43–230.81)		2.74 (0.96–5.38)
SI			5.07 (1.42–18.16)		2.02 (1.23–3.54)
MI			*P* = 0.56		*P* = 0.45
		**Any ACPA positive**		**Any ACPA negative**	
**HLA-SE**	**Smoking**	**Cases/controls**	**OR (95% CI)**	**Cases/controls**	**OR (95% CI)**
None	Never	23/173	1.00 (Ref)	16/173	1.00 (Ref)
None	Ever	42/162	1.95 (1.12–3.39)	35/162	2.34 (1.25–4.38)
Any	Never	52/125	3.13 (1.82–5.38)	25/125	2.16 (1.11–4.22)
Any	Ever	88/92	7.19 (4.26–12.15)	53/92	6.23 (3.37–11.51)
**Interaction**					
AP			0.43 (0.37–0.52)		0.44 (0.35–0.56)
RERI			3.11 (0.77–6.90)		2.73 (0.21–6.81)
SI			2.01 (1.15–3.52)		2.09 (1.02–4.29)
MI			*P* = 0.65		*P* = 0.62

OR for the development of RA. Attributable proportion due to interaction (AP), relative excess due to interaction (RERI), synergy index (SI), and multiple interaction (MI). HLA-SE= HLA shared epitope, OR= odds ratio, CI= confidence intervals, anti-CCP2=anti-cyclic citrullinated peptide antibodies, anti CEP-1/Eno5-21= antibodies against α-Enolase, anti-citC1359-369= antibodies against collagen type II aa 359-369 with two citrulline residues, anti- Fibα580-600=antibodies against fibrinogen (Fib) Fibα580-600, anti-Fibα561-583= antibodies against Fibα561-583, anti- Fibβ62-81a(72)=antibodies against Fibβ62-81a(72), anti-Fibβ62-81b(74)= antibodies against Fibβ62-81b(74), anti-Fibβ36-52= antibodies against Fibβ36-52, anti- CCP-1/Fil307-324= antibodies against filaggrin, anti- Vim60-75= antibodies against vimentin (Vim)60-75, anti-Vim2-17= antibodies against Vim 2-17, ACPA=anti-citrullinated peptide/protein antibody.

## Discussion

In this study blood samples that had been identified within population surveys from individuals years before they reported any symptoms of a subsequent joint disease were included. These samples together with samples from controls were analysed for different ACPA specificities in relation to genetic and environmental determinants. This is the first study where the relationships between the presence of a number of different ACPA besides anti-CCP2 antibodies, and HLA-SE and smoking have been analysed years before onset of symptoms.

In this study we have analysed the importance of HLA-SE and smoking for the development of ACPA and of the combination of these three risk factors for disease development. From this study we can conclude that the contribution of HLA-SE alleles and smoking prior to RA increased closer to disease onset and were moderate, but in combination with the most prevalent antibodies (CEP-1/Eno5-21, Fibß36-52, and CCP-1/Fil307-324) aside from anti-CCP2 antibodies, the relative risk for disease development was highly increased. We also found that the relationships between HLA-SE, smoking, and the different ACPA specificities showed variable patterns in individuals before they had developed symptoms of RA. Furthermore the presence of HLA-SE and smoking were independent from ACPA and anti-CCP2 antibodies as risk factors for disease development.

Overall, the conclusions are that in individuals who will subsequently develop RA, HLA-DRB1* SE alleles and smoking were associated with the development and increase of specific ACPA reactivities, although to a variable extent. The presence of various ACPA specificities and of anti-CCP2 antibodies was, to a moderate degree, associated with having HLA-SE and/or being an ever-smoker. These relationships are not as evident as has been shown in patients with manifest RA [6,7], probably as a result of lower frequencies of the various antibodies before disease onset compared with after the disease has developed. The frequencies of several of the different ACPA gradually increased as the disease developed (the closer to onset of RA that the blood was sampled) as previously shown [5], whilst obviously being HLA-SE allele-positive or an ever-smoker gave similar results irrespective of time. Considering the combination of HLA-SE and smoking with the development of the various antibodies, we could not show that HLA-SE or smoking alone was significant for the early development and first appearance of many of the antibodies when all individuals, irrespective of pre-dating time, were included. However, when including only samples ≤10.5 years prior to symptom onset of RA (when the concentration and frequencies of certain antibodies were increasing), the combination of smoking and HLA-SE was significantly associated with antibodies against several of the citrullinated peptides. Furthermore, most remained significant at diagnosis, such as antibodies against CCP2, CEP-1/Eno5-21, citC1359-369, Fibß62-81b, and Fibß36-52. The effect of HLA-SE alleles and smoking seemed to be of more importance when approaching disease onset when the frequencies of the different antibodies were increasing. Also, higher levels of anti-CCP2, −CEP-1/Eno5-21 and -Fibß62-81b antibodies, respectively (three times above the cutoff level) were associated with having HLA-SE alleles. These results could indicate that HLA-SE and/or smoking is of importance for expansion of some of the antibody specificities, but not for others. Thus, these antibodies could have a more significant role in the pathogenesis of the development of the disease, whilst the presence of the primary antibodies could result from less specific responses.

From comparing the relationships between HLA-SE, smoking, and ACPA reactivities in sera obtained before and after disease onset, respectively, we observed that presence of HLA-SE was associated with a conversion from seronegativity to seropositivity, occurring between the time point of sampling before onset of disease and the time point of sampling after the disease was developed. These data which indicate that HLA-SE is a major factor in determining whether an ACPA-positive individual will develop RA, are in accordance with the recent study of ACPA reactivities in twins with ACPA without disease and twins with ACPA and RA, which showed that twins with ACPA and RA were more frequently HLA-SE positive compared with twins with ACPA without RA [11]. The interaction analyses between HLA-SE and smoking was particularly strong in individuals positive for antibodies against CCP2, Fibß36-52, CEP-1/Eno5-21, and CCP-1/Fil307-324. An interaction was also seen between HLA-SE and smoking irrespective of the presence of any ACPA. Due to the resulting low number of individuals in each subgroup when stratified for number of HLA-SE alleles, we were unable to analyse for interaction based on the number of alleles.

The interaction between risk factors (HLA-SE alleles, smoking, and ACPA) for disease progression is complex. Since all of the cases included in this study were identified as having subsequently developed RA, it is difficult to generalize the results of the relationships between the factors. Interaction between HLA-SE and smoking predating disease onset has been shown in the study by Karlson *et al*. [12], and also in patients with early RA [7,21]. In the study by Karlson *et al*., the data was stratified for rheumatoid factor seropositivity, reaching the highest risk for heavy smokers with double copies of HLA-SE (OR = 7.47, 95% CI: 2.77 to 20.11) [12]. In another study by Arkema *et al*. [13] on 192 preclinical RA cases, the frequency of positivity for any ACPA, including 18 specificities, was 25% and 6% in controls. In that study, the combination of HLA-SE and ACPA positivity compared with those negative for HLA-SE and ACPA yielded an OR of 33.3 (95% CI: 11.1 to 99.6), when restricting the analyses to those with blood sampled less than five years before the onset of disease [13]. Our data showed a similar high OR for the combination of HLA-SE and anti-CCP2 antibodies compared with being HLA-SE and anti-CCP2 negative; although the present data included all samples (median of 4.6 (IQR: 2.0 to 9.7) years) irrespective of reducing the pre-dating time to less than five years before onset of symptoms. The combinations of the three most prevalent ACPA specificities (CEP-1/Eno5-21, Fibß36-52, and CCP-1/Fil307-324) together with smoking and HLA-SE yielded a very high risk for development of RA relative to the reference of being negative for all three factors. The combination of the three most frequently occurring ACPA together with anti-CCP2 antibodies in ever -smokers with HLA-SE alleles gave the highest relative risk (OR = 105.7).

The estimated risks in some of the analyses should be interpreted in the context of small sample sizes with resulting large CIs. There are no clear difference in the risk ratios between the ACPAs appearing with the highest frequencies (CEP-1/Eno5-21, Fibß36-52, and CCP-1/Fil307-324), although each of them was clearly lower when compared with anti-CCP2. Analysing a combination of the three yielded similar results compared with that for anti-CCP2, and a very high relative risk was found when analysing the triple combination together with anti-CCP2.

We are unable to confirm the results by Lundberg *et al*., who reported the most pronounced effects of reactivity to CEP-1/Eno5-21 in early RA patients when compared with that for Fibß36-52, CCP-1/Fil307-324, and Vim60-75 [9]. However, the present study represented individuals before the onset of symptoms of RA, of whom all will develop the disease, and it was not powered to compare whether the achieved results differed from those in early RA presented by Lundberg *et al*. [9]. Also, in the study by Lundberg *et al*. the different ACPA specificities were analysed using ELISAs, without subtraction of the arginine reactivities from the analyses [9].

## Conclusions

From this study we can conclude that the interaction between risk factors for antibody development (HLA-SE alleles, smoking, and ACPA) and disease progression is complex. The development of increasing frequencies of positivity of ACPA is related to the presence of HLA-SE and being an ever-smoker. All three factors increase the risk of disease development separately, and also by an interaction between HLA-SE and smoking.

## Additional file

Additional file 1: Table S1. Results of multiple regression analyses (including antibodies, smoking, and HLA-SE, respectively) for development of RA in individuals before the onset of any symptoms of disease.

## Abbreviations

ACPA: anti-citrullinated protein antibodies
anti-CCP: anti-cyclic citrullinated peptide antibodies
AP: attributable proportion
CEP-1/Eno5-21: α-enolase
CI: confidence interval
ELISA: enzyme-linked immunosorbent assay
Fib: fibrinogen
Filaggrin: CCP-1/Fil307-324
HLA-SE: human leukocyte antigen-shared epitope
OR: odds ratio
*PTPN22*: protein tyrosine phosphatase non receptor type 22
RA: rheumatoid arthritis
RERI: relative excess risk due to interaction
SI: synergy index
citC1359-369: triple helical collagen type II, peptide containing the triple helical aa 359-369 with two citrulline residues
Vim: vimentin
