# Supplementary material for: Associations of antibodies against citrullinated peptides with human leukocyte antigen-shared epitope and smoking prior to the development of rheumatoid arthritis
Source: Arthritis Res Ther. 2015 May 20;17(1):125. doi: 10.1186/s13075-015-0638-x (PMC4438519; doi:10.1186/s13075-015-0638-x)
Supplement: Additional file 1: Table S1. — Results of multiple regression analyses (including antibodies, smoking, and HLA-SE, respectively) for development of RA in individuals before the onset of any symptoms of disease. [file 13075_2015_638_MOESM1_ESM.docx]

Table S1. Results of multiple regression analyses (including antibodies, smoking

and HLA-SE, respectively) for development of RA in individuals before the onset of any

symptoms of disease.

| **Antibodies** | **OR (95% CI)**  **antibody** | **OR (95% CI)**  **smoking** | **OR (95% CI)**  **HLA-SE** |
| --- | --- | --- | --- |
| **Anti-CCP2** | 19.41 (10.63, 35.45) | 2.02 (1.47, 2.77) | 2.59 (1.89, 3.54) |
| **Anti-CEP-1** | 5.12 (3.33, 7.88) | 2.21 (1.63, 2.98) | 2.93 (2.17, 3.96) |
| **Anti-CitC1** | 3.01 (1.76, 5.16) | 2.3 (1.71, 3.09) | 3.05 (2.27, 4.09) |
| **AntiFibα580-600 (591)** | 1.53 (0.89, 2.65) | 2.37 (1.77, 3.18) | 3.10 (2.32, 4.16) |
| **Anti-Fibα561-583(573)** | 1.98 (1.16, 3.38) | 2.37 (1.76, 317) | 3.10 (2.32, 4.15) |
| **Anti-Fibβ62-81a( 72)** | 3.71 (2.04, 6.72) | 2.52 (1.87, 3.38) | 2.96 (2.21, 3.97) |
| **Anti-Fibβ62-81b(74)** | 8.85 (4.36, 17.97) | 2.24 (1.66, 3.03) | 2.85 (2.11, 3.85) |
| **Anti-Fibβ36-52** | 5.82 (3.79, 9.96) | 2.28 (1.68, 3.09) | 2.98 (2.20, 4.04) |
| **Anti-Fil307-324** | 10.63 (6.04, 18.72) | 2.34 (1.72, 3.18) | 2.75 (2.03, 3.74) |
| **Anti-Vim60-75** | 2.29 (1.37, 3.81) | 2.46 (1.84, 3.30) | 3.10 (2.32, 4.15) |
| **Anti-Vim2-17** | 1.49 (0.79, 2.81) | 2.39 (1.78, 3.21) | 3.12 (2.34, 4.17) |
| **Any ACPA** | 3.99 (2.94, 5.42) | 2.34 (1.72, 3.18) | 2.97 (2.18, 4.03) |
